# Engineering Modification Enhances the Antibacterial Activity of Yak Gastric Lysozyme and Its High-Level Expression in *Pichia pastoris*

**DOI:** 10.3390/ijms27219403

**Published:** 2026-10-22

**Authors:** Bing Bing Fan, An Rong Zhang, Zhen Long Wu, Ying Yang

**Affiliations:** College of Animal Science and Technology, China Agricultural University, Beijing 100083, China; bingbingfan0305@163.com (B.B.F.); b20213040357@cau.edu.cn (A.R.Z.)

**Keywords:** yak gastric lysozyme, *Pichia pastoris*, antibacterial activity, hydrophobic peptide modification, expression optimization

## Abstract

To broaden the antibacterial spectrum of yak gastric lysozyme (YGL) against Gram-negative bacteria and improve its secretory expression in *Pichia pastoris*, this study employed a combined strategy of functional short-peptide fusion and host engineering. Four hydrophobic peptides, FFVAP, GVSLANWVCLAK, VVYP, and AWVAWK, were individually fused to the C-terminus of YGL, and the enzymatic activity and antibacterial performance of the modified proteins were compared. AW-YGL was subsequently selected for sequential optimization of the host strain, secretion signal peptide, endoplasmic-reticulum-associated molecular chaperones, and clustered regularly interspaced short palindromic repeats/CRISPR-associated protein 9 (CRISPR/Cas9)-mediated host-gene knockout, followed by physicochemical stability evaluation of the optimized product. The results showed that AWVAWK modification produced the most pronounced enhancement of YGL activity against Gram-negative bacteria. AW-YGL exhibited inhibition rates of 65%, 68%, and 67% against *Escherichia coli*, *Pseudomonas aeruginosa*, and *Salmonella enteritidis*, respectively. During expression-system optimization, the GS115 host significantly improved AW-YGL secretion, with total enzyme activity and specific activity in the culture supernatant reaching 3.1 × 10^5^ U/L and 983 U/mg, respectively. Replacement with the HSS signal peptide further increased total enzyme activity to 4.3 × 10^5^ U/L and specific activity to 1264 U/mg. Co-expression of the endoplasmic reticulum molecular chaperone binding immunoglobulin protein (BIP) further promoted functional AW-YGL expression, increasing the specific activity to 1560.3 U/mg. On this basis, knockout of the cell-wall-related gene FKS1 further increased the total enzyme activity in the culture supernatant to 6.9 × 10^5^ U/L and the specific activity to 1638.3 U/mg, indicating that stepwise optimization of the signal peptide, folding assistance, and host-cell engineering effectively enhanced AW-YGL secretory expression and enzymatic activity. The optimized AW-YGL retained relatively high activity over defined pH and temperature ranges and showed tolerance to several metal ions and digestion-related proteases. Collectively, C-terminal fusion with hydrophobic short peptides enhanced the inhibitory activity of YGL against Gram-negative bacteria, while the combined strategies of signal-peptide optimization, folding assistance, and cell-wall engineering further improved its secretory expression in *P. pastoris*. These findings provide an experimental basis for the engineering and scalable production of novel broad-spectrum lysozymes.

## 1. Introduction

The widespread use of antibiotics in clinical medicine, animal production, and the food industry has intensified the problem of bacterial antimicrobial resistance, making it a major global public-health challenge. Gram-negative bacteria are particularly difficult to control because lipopolysaccharide (LPS) in their outer membrane forms an intrinsic permeability barrier that restricts the entry of many conventional antimicrobial agents. Therefore, the development of natural antimicrobial molecules with novel modes of action, favorable safety profiles, and a low propensity to induce resistance has become an important research priority.

Lysozymes are natural antimicrobial proteins widely distributed in animals, plants, and microorganisms. They cause bacterial lysis by hydrolyzing the β-1,4 glycosidic bond in cell-wall peptidoglycan. Owing to their natural origin, favorable safety profile, and distinctive mechanism of action, lysozymes have been widely used in food preservation, biomedicine, and animal nutrition. However, conventional lysozymes, such as hen egg-white lysozyme, are generally more active against Gram-positive bacteria, whereas their activity against Gram-negative bacteria is limited by the outer-membrane barrier, which substantially restricts their broader application [1,2,3,4].

Mammalian gastric lysozymes represent a functional class of lysozymes that have adapted during evolution to the specialized gastric environment. Compared with conventional lysozymes derived from body fluids or secretions, gastric lysozymes are chronically exposed to acidic conditions and protease-rich digestive environments and therefore generally exhibit favorable acid stability and protease resistance [5,6].

Yaks inhabit cold, high-altitude environments, and their stomachs contain complex microbial ecosystems under tightly regulated digestive conditions. Yak gastric lysozyme (YGL), as an important component of the ruminant gastric defense system, may possess environmental adaptability and structural stability, making it a promising scaffold for the development of novel antimicrobial proteins.

However, as with many natural lysozymes, the activity of YGL against Gram-negative bacteria remains restricted by the bacterial outer membrane. Thus, enhancing membrane interaction or penetration while preserving the native catalytic activity of YGL is an important strategy for broadening its antibacterial spectrum.

Numerous studies have shown that natural antimicrobial peptides are often enriched in hydrophobic amino acids and aromatic residues, and their antibacterial effects depend on both electrostatic and hydrophobic interactions with bacterial membrane lipids. Hydrophobic residues such as tryptophan (Trp), phenylalanine (Phe), valine (Val), and leucine (Leu) can enhance peptide association with membrane interfaces, promote membrane perturbation, and thereby improve antibacterial activity [7,8,9,10,11].

Based on these structure–activity relationships, four short peptides with distinct hydrophobicity, aromatic-residue composition, and net-charge characteristics were selected as candidate functional modules. Among them, AWVAWK is an antimicrobial hexapeptide identified by enzymatic hydrolysis of porcine lysozyme and has been reported to possess activity against Gram-negative bacteria as well as potential membrane-penetrating properties [12]. FFVAP, GVSLANWVCLAK, and VVYP were included as candidate peptides with different hydrophobic and aromatic-residue features. This design enabled comparison of how different peptide physicochemical properties affect the catalytic activity and anti-Gram-negative performance of YGL.

Because the N-terminal region of lysozyme is closely associated with secretory processing and protein folding, the selected hydrophobic peptides were fused to the C-terminus of YGL. This strategy was intended to preserve the catalytic architecture of YGL while conferring additional membrane-interacting properties, thereby enhancing its antibacterial activity against Gram-negative bacteria [13,14,15].

*Pichia pastoris* is widely used for recombinant protein production because of its capacity for high-cell-density cultivation, well-established secretory expression system, and efficient protein-folding machinery. Nevertheless, heterologous protein production can still be constrained by inefficient secretion, protein misfolding, and endoplasmic reticulum (ER) stress [16,17,18,19,20,21].

Yak gastric lysozyme is a secreted glycoprotein, and its biological properties may be influenced by post-translational modifications during heterologous expression. Similar to other eukaryotic secreted proteins, potential post-translational modifications (PTMs) of YGL in *P. pastoris* may include N-linked glycosylation, disulfide bond formation, and proteolytic processing during secretion. Alterations in glycosylation-related pathways or ER-associated processing may therefore affect protein folding, maturation, secretion efficiency, and enzymatic performance.

Accordingly, a multilevel host-engineering strategy was further applied to improve AW-YGL expression. First, secretion efficiency was optimized by screening signal peptides from different sources. Second, the ER-associated factors HAC1, CPR5, PDI, ERO1, BIP, and AFT1 were co-expressed to enhance protein folding and ER processing. In addition, clustered regularly interspaced short palindromic repeats/CRISPR-associated protein 9 (CRISPR/Cas9)-mediated knockout of MNN1 and FKS1 was performed to modulate cell-wall structure and glycosylation-related processes and thereby improve recombinant protein expressionMNN1 and FKS1 were selected as engineering targets because both genes are associated with important cellular processes that may influence heterologous protein production in *Pichia pastoris*. MNN1 encodes an α-1,6-mannosyltransferase involved in outer-chain mannose extension during protein glycosylation, whereas FKS1 encodes a β-glucan synthase involved in cell wall β-glucan formation and cell wall organization. Therefore, modification of these pathways may influence recombinant protein processing, secretion efficiency, or extracellular accumulation [22,23,24,25].

In summary, this study integrated functional-module design, expression-system optimization, and host-cell engineering to construct a recombinant lysozyme with enhanced inhibitory activity against Gram-negative bacteria, providing a theoretical and experimental basis for the rational engineering and further investigation toward scalable production of natural antimicrobial proteins.

## 2. Results

### 2.1. Fusion of Hydrophobic Short Peptides Enhances the Inhibitory Activity of Yak Gastric Lysozyme Against Gram-Negative Bacteria

Yak gastric lysozyme (YGL) exhibits favorable lysozyme activity, but its inhibitory activity against Gram-negative bacteria is restricted by the outer-membrane barrier. To improve the antibacterial properties of YGL, four short peptides with different hydrophobicity profiles—FFVAP (FF), GVSLANWVCLAK (GV), VVYP (VV), and AWVAWK (AW)—were individually fused to the C-terminus of YGL to generate four modified lysozymes: FF-YGL, GV-YGL, VV-YGL, and AW-YGL.

**Figure 1:**
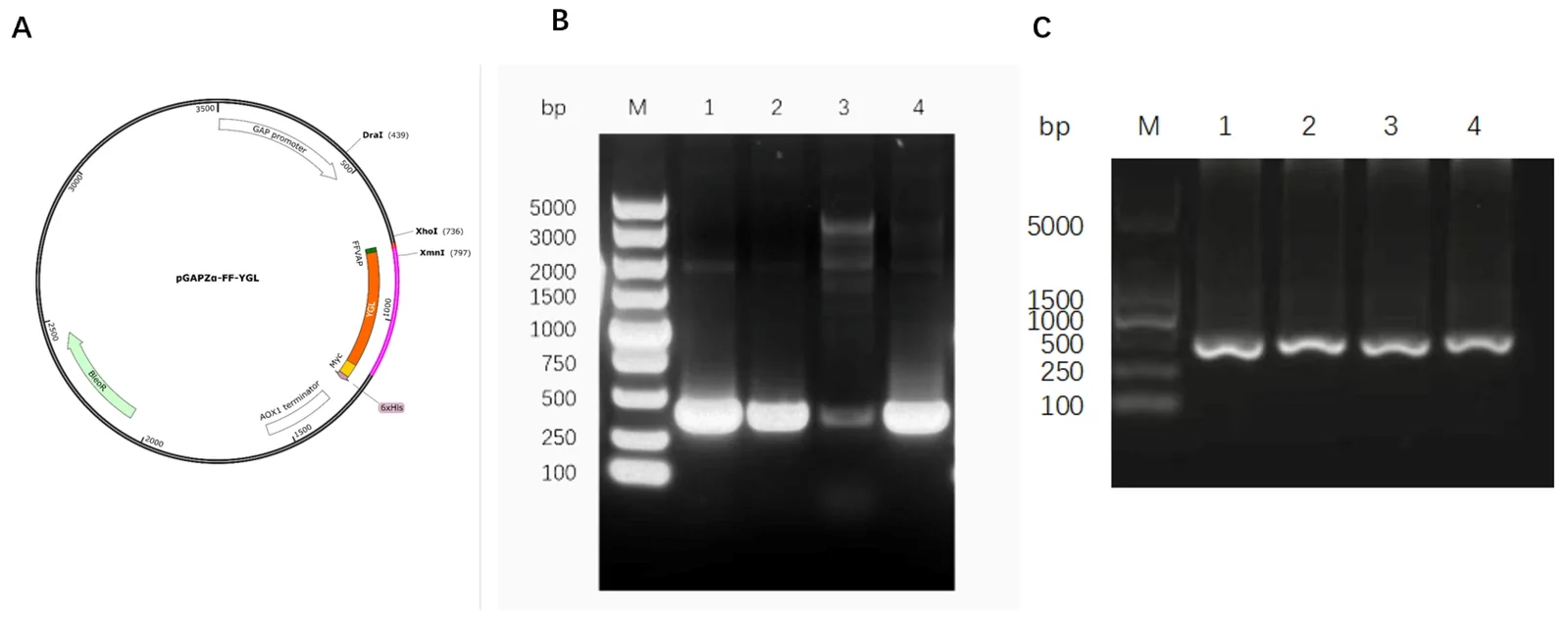
Construction of the pGAPZα-FF-YGL recombinant expression vector and polymerase chain reaction (PCR) identification of recombinant *P. pastoris* strains. (**A**) Map of the pGAPZα-FF-YGL recombinant expression vector; (**B**) PCR identification of recombinant plasmids. M, DNA marker; lanes 1–4, FF-YGL, GV-YGL, VV-YGL, and AW-YGL recombinant plasmid clones, respectively; (**C**) genomic PCR identification of recombinant *P. pastoris* transformants. M, DNA marker; lanes 1–4, FF-YGL, GV-YGL, VV-YGL, and AW-YGL, respectively.

To construct the recombinant expression vector for hydrophobic peptide–YGL fusion proteins, the target gene encoding the peptide-YGL fusion sequence was inserted into the pGAPZα expression vector under the control of the GAP promoter. The recombinant plasmid contained the α-factor secretion signal sequence, the peptide-YGL coding region, and the C-terminal His tag for subsequent protein purification (Figure 1A). The recombinant vectors were transformed into *E. coli* TOP10 cells. Colony polymerase chain reaction (PCR) amplification and sequencing showed that the hydrophobic-peptide-fused YGL gene fragments were successfully constructed, producing amplicons of approximately 400 bp, consistent with the expected size (Figure 1B). The recombinant plasmids were linearized, purified, and transformed into *P. pastoris* X-33 for expression analysis. Yeast colony PCR and sequencing produced amplicons of approximately 400 bp, consistent with the expected size, confirming successful transformation and genomic integration of the recombinant plasmids (Figure 1C).

To investigate whether hydrophobic peptide fusion affected recombinant YGL production and enzymatic properties, four hydrophobic peptides (FFVAP, GVSLANWVCLAK, VVYP, and AWVAWK) were fused to the C-terminus of YGL, and the corresponding recombinant proteins were expressed in *Pichia pastoris* X-33.

**Figure 2:**
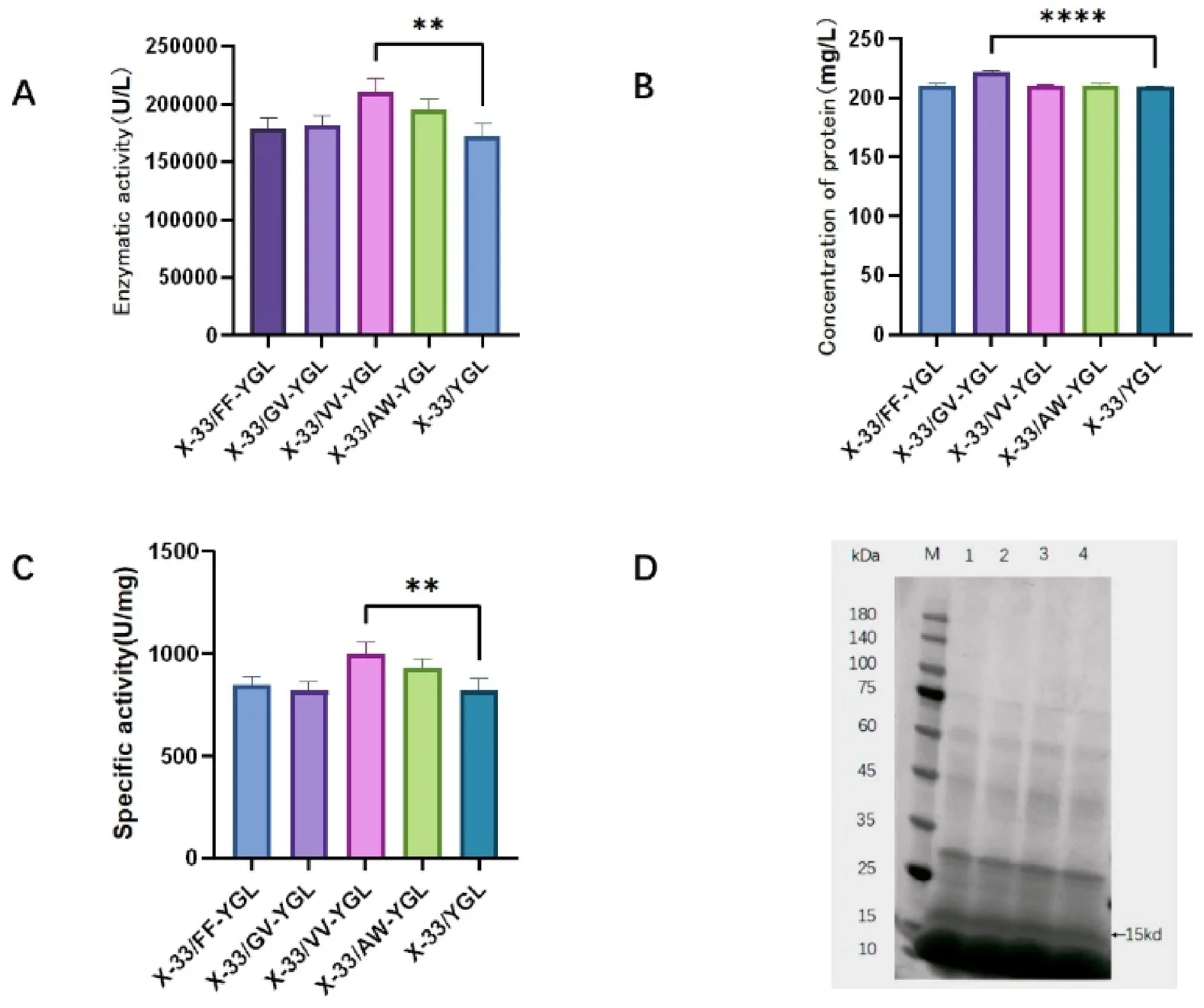
Effects of hydrophobic short-peptide fusion on the expression level and enzymatic properties of yak gastric lysozyme (YGL). (**A**) Lysozyme activity in fermentation supernatants of recombinant YGL strains fused to different hydrophobic peptides. X-33/FF-YGL, X-33/GV-YGL, X-33/VV-YGL, and X-33/AW-YGL denote YGL-expressing strains fused with FF, GV, VV, and AW, respectively, whereas X-33/YGL is the non-fused control strain. Different peptide fusions affected YGL enzyme activity to varying degrees, with VV-YGL showing the highest activity. (**B**) Total AW-YGL concentration after purifications in fermentation supernatants of the different fusion proteins. Hydrophobic-peptide fusion had little effect on secreted protein levels, and no significant differences were observed among the groups. (**C**) Specific activities of the fusion proteins. VV-YGL showed the highest specific activity and was higher (with the corresponding numerical values and statistical results provided) than the non-fused YGL control, indicating that hydrophobic-peptide fusion may affect not only expression but also YGL folding or maintenance of a favorable active conformation. (**D**) sodium dodecyl sulfate-polyacrylamide gel electrophoresis (SDS-PAGE) analysis of the fusion proteins. M, protein molecular-weight marker; lanes 1–4, FF-YGL, GV-YGL, VV-YGL, and AW-YGL recombinant protein samples, respectively. A target band of approximately 15 kDa was detected for all fusion proteins, consistent with the theoretical molecular weight of YGL and further indicating the presence of a recombinant protein band consistent with the expected molecular weight. Data are presented as mean ± SD (*n* = 3). * indicates statistical significance (** *p* < 0.01, **** *p* < 0.0001).

Sodium dodecyl sulfate-polyacrylamide gel electrophoresis (SDS-PAGE) analysis of fermentation supernatants revealed apparent protein bands around 15 kDa for the recombinant proteins, which were consistent with the theoretical molecular weight of YGL (Figure 2D). These results suggested the presence of recombinant proteins with the expected molecular size in the culture supernatants.

Enzymatic activity analysis showed that hydrophobic peptide fusion altered the activity of recombinant YGL to different extents (Figure 2A). Among the tested constructs, VV-YGL exhibited the highest enzymatic activity (2.1 × 10^5^ U/L), representing an approximately 21.1% increase compared with the non-fused YGL control (1.7 × 10^5^ U/L, *p* < 0.01). AW-YGL also showed increased enzymatic activity (1.9 × 10^5^ U/L) compared with YGL, although the difference was less pronounced.

The purified recombinant protein concentrations were comparable among the different constructs, ranging from 209.7 to 221.3 mg/L (Figure 2B), indicating that hydrophobic peptide fusion did not markedly affect the amount of purified protein obtained under the tested conditions.

To further evaluate the influence of peptide fusion on catalytic efficiency, the specific activity was calculated based on enzymatic activity and purified protein concentration. VV-YGL exhibited the highest specific activity (1000.3 U/mg), which was approximately 21.8% higher than the YGL control (821.0 U/mg, *p* < 0.01) (Figure 2C). AW-YGL also showed an increased specific activity (926.4 U/mg) compared with unmodified YGL.

These results indicate that hydrophobic peptide fusion primarily influenced the functional activity of recombinant YGL rather than protein accumulation, with VVYP and AWVAWK fusion showing greater enhancement of enzymatic performance.

**Figure 3:**
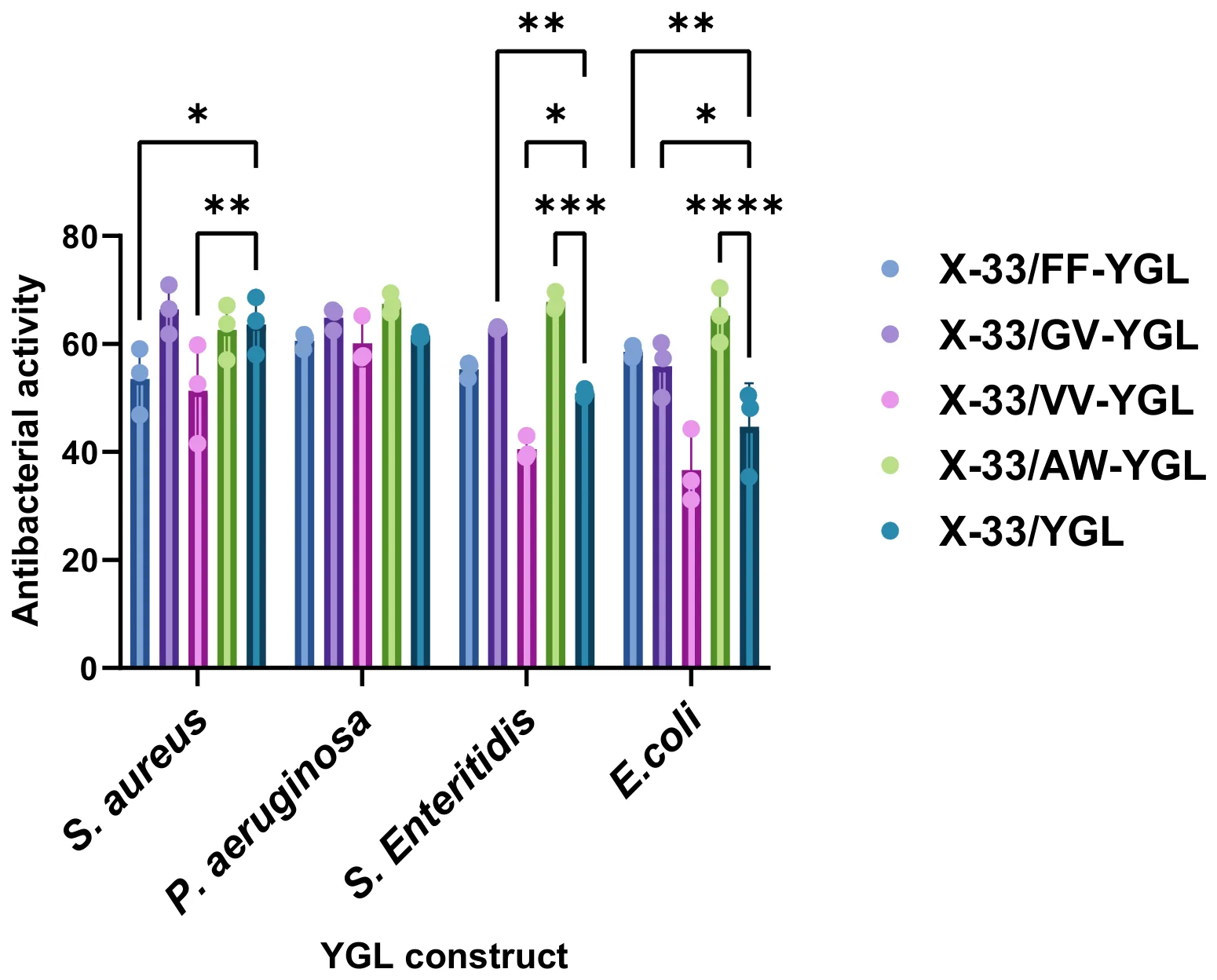
Effects of hydrophobic short-peptide fusion on the antibacterial activity of yak gastric lysozyme (YGL). Antibacterial activity was evaluated against *Staphylococcus aureus*, *Pseudomonas aeruginosa*, *Salmonella enteritidis*, and *Escherichia coli* using recombinant YGL fused with FF, GV, VV, or AW peptides. X-33/YGL was used as the non-fused control. Data are presented as mean ± SD (*n* = 3). Statistical significance was evaluated by two-way analysis of variance (ANOVA) with hydrophobic peptide construct and bacterial species as the two factors, followed by Dunnett’s multiple-comparisons test using X-33/YGL as the reference group. * *p* < 0.05, ** *p* < 0.01, *** *p* < 0.001, **** *p* < 0.0001.

The antibacterial activities of the modified YGL proteins were subsequently evaluated. Hydrophobic-peptide fusion markedly enhanced YGL inhibition of Gram-negative bacteria (Figure 3). Compared with unmodified YGL, AW-YGL showed enhanced antibacterial activity against several Gram-negative bacteria. Specifically, AW-YGL exhibited inhibition rates of 67.5 ± 1.85%, 67.8 ± 1.70%, and 65.3 ± 5.05% against *P. aeruginosa*, *S. enteritidis*, and *E. coli*, respectively, compared with 61.6 ± 0.60%, 50.9 ± 0.78%, and 44.7 ± 8.21% for YGL. The increases against *S. enteritidis* and *E. coli* were statistically significant (*p* < 0.001 and *p* < 0.01, respectively). In contrast, AW-YGL showed no significant difference compared with YGL against the Gram-positive strain *S. aureus* (62.7 ± 5.18% vs. 63.7 ± 5.31%), indicating that hydrophobic peptide fusion preferentially enhanced the antibacterial activity of YGL against Gram-negative bacteria.

These results demonstrate that hydrophobic short-peptide fusion can effectively enhance the inhibitory activity of YGL against Gram-negative bacteria, with AW-YGL showing the strongest antibacterial effect. VV-YGL displayed the highest enzyme activity and specific activity, whereas AW-YGL combined relatively high catalytic activity with the strongest anti-Gram-negative activity. The difference between enzymatic activity and antibacterial performance suggests that antibacterial efficacy of recombinant YGL is not solely determined by catalytic activity. Other factors, including peptide-mediated interactions with bacterial surfaces and membrane permeability changes, may also contribute to the observed antibacterial activity. Further studies are required to clarify the detailed mechanism. Therefore, AW-YGL was selected for subsequent expression optimization.

### 2.2. Host-Strain Optimization Promotes AW-YGL Expression

**Figure 4:**
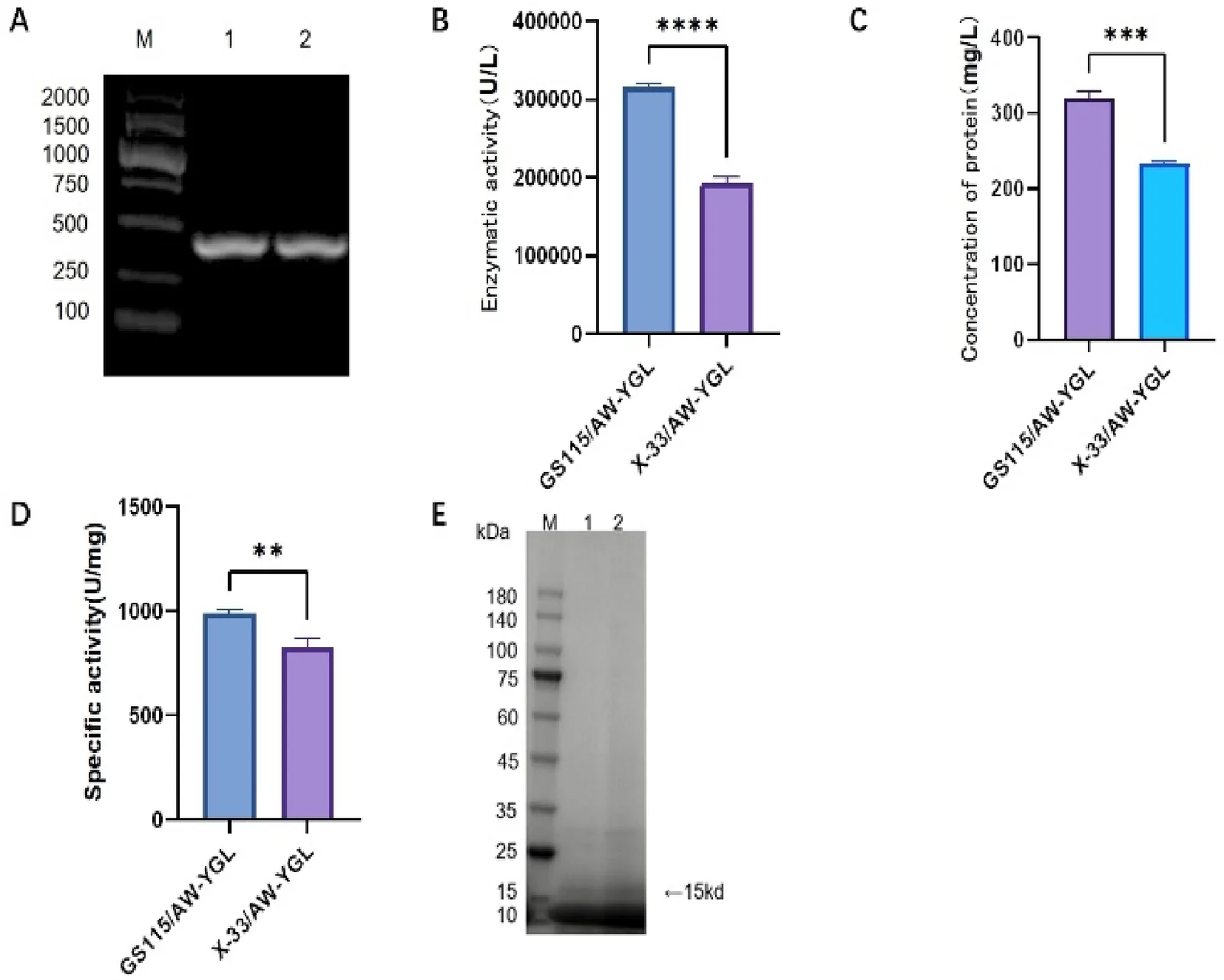
Effects of different *P. pastoris* hosts on the expression and enzymatic properties of AW-YGL. (**A**) Colony polymerase chain reaction (PCR) identification of recombinant *P. pastoris* strains. M, DNA marker; lane 1, GS115/AW-YGL; lane 2, X-33/AW-YGL. PCR products of the expected size confirmed successful genomic integration of the AW-YGL expression cassette in both hosts. (**B**) Enzyme activity of AW-YGL expressed in different *P. pastoris* hosts. GS115/AW-YGL and X-33/AW-YGL denote recombinant strains expressing AW-YGL in GS115 and X-33, respectively. The lysozyme activity in the fermentation supernatant of GS115/AW-YGL was higher (with the corresponding numerical values and statistical results provided) than that of X-33/AW-YGL, demonstrating a marked host-dependent effect on AW-YGL expression. (**C**) Purified AW-YGL concentration of the two host systems. The GS115/AW-YGL group exhibited significantly greater protein secretion than the X-33/AW-YGL group, indicating that GS115 favored AW-YGL secretory expression. (**D**) Specific activity of AW-YGL produced by the two host systems. GS115/AW-YGL showed higher specific activity, suggesting that host optimization not only increased recombinant-protein output but also helped maintain an active YGL conformation. (**E**) Verification of AW-YGL expression. M, protein molecular-weight marker; lane 1, GS115/AW-YGL; lane 2, X-33/AW-YGL. sodium dodecyl sulfate-polyacrylamide gel electrophoresis (SDS-PAGE) showed an approximately 15 kDa target band in both host systems, consistent with the theoretical molecular weight of YGL and supporting the presence of recombinant AW-YGL. Data are presented as mean ± SD (*n* = 3). ** *p* < 0.01, *** *p* < 0.001, **** *p* < 0.0001.

To evaluate the influence of different *Pichia pastoris* host backgrounds on recombinant AW-YGL production, GS115/AW-YGL and X-33/AW-YGL strains were constructed and compared. SDS-PAGE analysis of fermentation supernatants showed an apparent protein band at approximately 15 kDa in both recombinant strains, consistent with the theoretical molecular weight of AW-YGL, indicating that AW-YGL was successfully secreted by both GS115 and X-33 hosts (Figure 4A).

Enzymatic activity analysis revealed that GS115/AW-YGL exhibited significantly higher activity than X-33/AW-YGL. The fermentation supernatant of GS115/AW-YGL showed an enzymatic activity of 3.1 × 10^5^ U/L, which was approximately 63.2% higher than that of X-33/AW-YGL (1.9 × 10^5^ U/L, **** *p* < 0.0001) (Figure 4B).

To further compare recombinant protein production, purified AW-YGL concentrations were determined after protein purification. GS115/AW-YGL produced 320 mg/L purified recombinant protein, which was approximately 39.1% higher than X-33/AW-YGL (230 mg/L, *** *p* < 0.001) (Figure 4C). These results indicate that the GS115 background contributed to improved recombinant AW-YGL production.

Because differences in total enzyme activity may result from variations in protein yield, the specific activity of purified AW-YGL was further calculated. GS115/AW-YGL exhibited a specific activity of 983 U/mg, which was approximately 19.7% higher than that of X-33/AW-YGL (821 U/mg, ** *p* < 0.01) (Figure 4D). This finding suggests that host background affected not only recombinant protein production but also the functional activity of the obtained AW-YGL protein.

SDS-PAGE analysis of purified AW-YGL samples revealed a predominant protein band at approximately 15 kDa in both recombinant strains, which was consistent with the theoretical molecular weight of AW-YGL (Figure 4E).

Although histidine auxotrophy is unlikely to be the primary factor affecting recombinant protein secretion under nutrient-rich conditions, differences in the genetic background and physiological state of GS115 and X-33 may contribute to the observed expression variation. These results indicate that GS115 was superior to X-33 in both AW-YGL secretory expression and activity retention; therefore, GS115 was selected as the host for subsequent AW-YGL expression optimization.

### 2.3. Signal-Peptide Optimization Enhances AW-YGL Secretion in Pichia pastoris

Because protein-secretion efficiency is an important factor limiting product yield in *P. pastoris*, different secretion signal peptides were evaluated to optimize AW-YGL expression.

**Figure 5:**
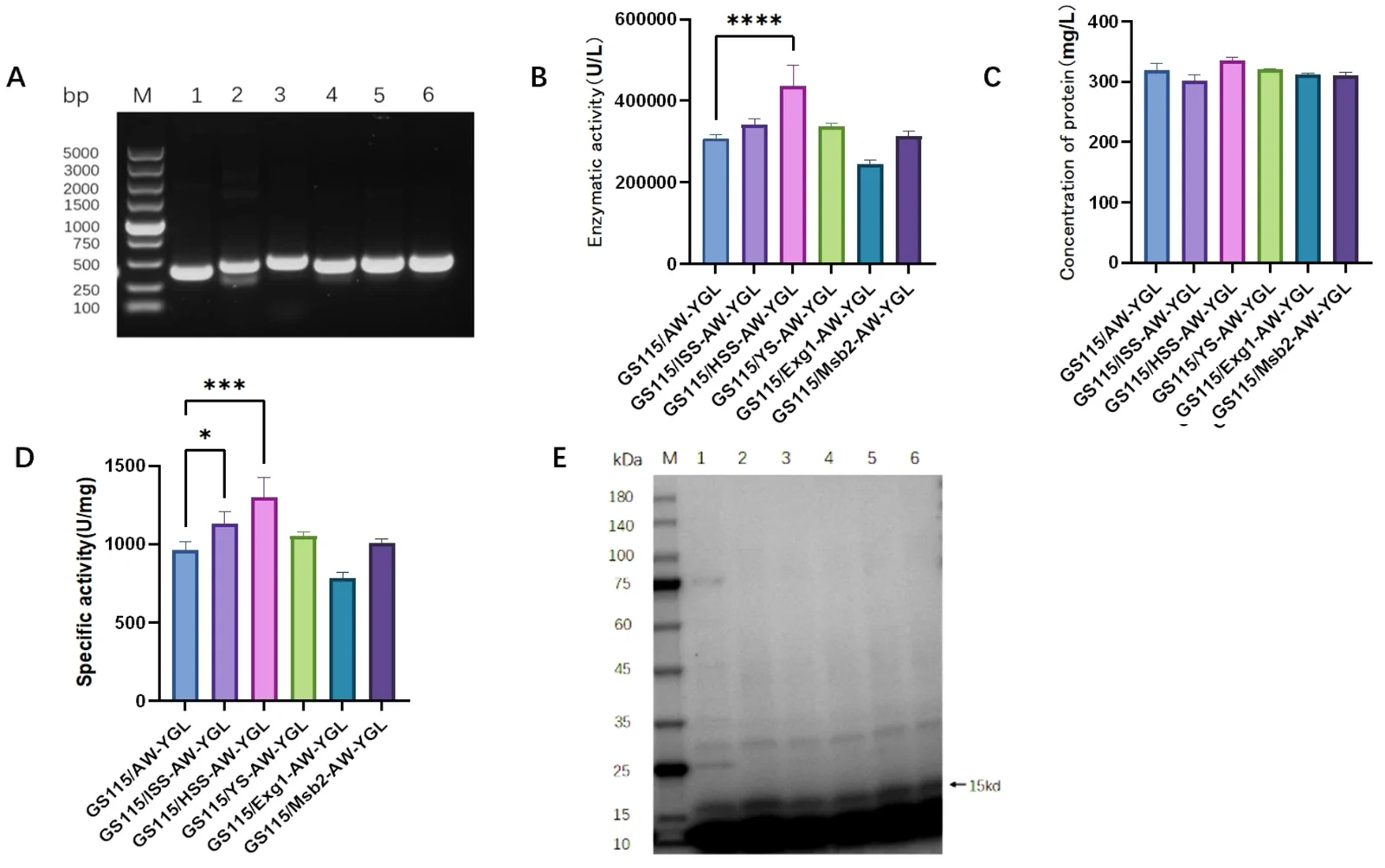
Effects of different signal peptides on recombinant AW-YGL expression and enzymatic properties in *P. pastoris***.** (**A**) polymerase chain reaction (PCR) verification of target genes in different recombinant *P. pastoris* strains. M, DNA molecular-weight marker; lanes 1–6, different recombinant strains. (**B**) AW-YGL enzyme activity in fermentation supernatants of the different recombinant strains. (**C**) purified AW-YGL concentrations. (**D**) Specific activity of AW-YGL. (**E**) sodium dodecyl sulfate-polyacrylamide gel electrophoresis (SDS-PAGE) analysis of fermentation supernatants from different recombinant strains. M, protein molecular-weight marker; lanes 1–6, the control strain followed by strains carrying the ISS, HSS, YS, Exg1, and Msb2 signal peptides, respectively. The arrow indicates AW-YGL at approximately 15 kDa. Data are presented as mean ± SD. * *p* < 0.05, *** *p* < 0.001, **** *p* < 0.0001.

The native α-factor signal peptide was replaced with HSS, ISS, YS, Exg1, or Msb2 to construct AW-YGL strains carrying different secretion signals. PCR analysis showed amplification products of the expected size for all signal-peptide fusion constructs, confirming successful recombinant-vector construction (Figure 5A).

After fermentation, the expression levels of the different signal-peptide strains were compared. The signal peptides had clear differential effects on AW-YGL secretion, with HSS producing the most pronounced enhancement. Among the tested signal peptides, only the optimal signal peptide fusion significantly increased AW-YGL production and specific activity, whereas the effects of other signal peptides were not statistically significant.

Enzyme-activity analysis showed that the total lysozyme activity in the culture supernatant of GS115/HSS-AW-YGL reached 4.3 × 10^5^ U/L, higher (with the corresponding numerical values and statistical results provided) than the 3.1 × 10^5^ U/L of the original AW-YGL strain (****, *p* < 0.0001) (Figure 5B). Its protein expression level reached approximately 320 mg/L (Figure 5C), which was markedly higher than that of the original strain.

Calculation of specific activity further showed that the HSS signal peptide promoted the accumulation of functional AW-YGL, with GS115/HSS-AW-YGL reaching 1264 U/mg, higher (with the corresponding numerical values and statistical results provided) than the control group (Figure 5D). SDS-PAGE analysis showed apparent protein bands around 15 kDa in all recombinant strains (Figure 5E).

Therefore, HSS was identified as the optimal signal peptide for improving AW-YGL secretory expression.

### 2.4. Co-Expression of Molecular Chaperones Enhances Heterologous AW-YGL Expression

**Figure 6:**
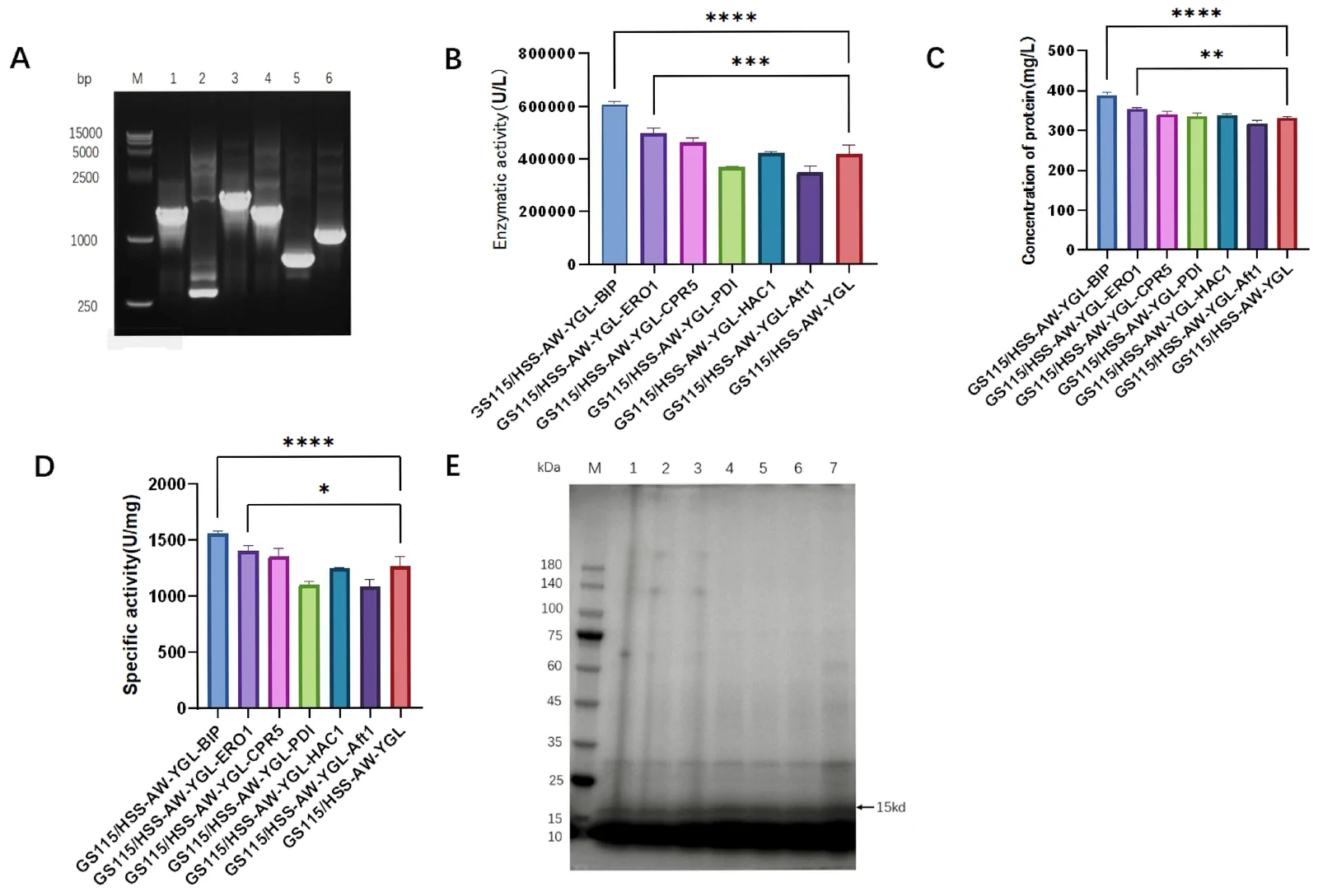
Effects of co-expression of different folding-assistance factors on AW-YGL expression. (**A**) Colony polymerase chain reaction (PCR) identification of recombinant strains carrying different folding-assistance factor expression constructs. M, DNA marker; lanes 1–6 represent recombinant strains carrying PDI, AFT1, BIP, ERO1, CPR5, and HAC1, respectively. PCR analysis confirmed successful genomic integration of all expression modules. (**B**) AW-YGL enzyme activity in the different folding-assistance co-expression systems. Using GS115/15H5-AW-YGL as the background strain, BIP, ERO1, CPR5, PDI, HAC1, and AFT1 co-expression systems were constructed. The BIP co-expression system exhibited the highest enzyme activity and was higher (with the corresponding numerical values and statistical results provided) than the control without an additional folding factor, indicating that BIP effectively promoted functional AW-YGL expression. (**C**) Purified AW-YGL concentrations in fermentation supernatants of the different co-expression systems. The different factors had showed only a limited change compared with the control effects on total secreted protein levels. (**D**) Specific activity of AW-YGL in the different co-expression systems. The BIP co-expression system exhibited relatively high specific activity, indicating that BIP-assisted expression was beneficial for YGL folding quality. (**E**) sodium dodecyl sulfate-polyacrylamide gel electrophoresis (SDS-PAGE) analysis of AW-YGL expression in the different co-expression systems. M, protein molecular-weight marker; lanes 1–7 correspond to the control and strains co-expressing PDI, AFT1, BIP, ERO1, CPR5, and HAC1, respectively. An approximately 15 kDa target band was detected in all groups, further supporting the presence of recombinant AW-YGL. Data are presented as mean ± SD (*n* = 3). * *p* < 0.05, ** *p* < 0.01, *** *p* < 0.001, **** *p* < 0.0001.

Protein folding efficiency is an important factor affecting heterologous protein production in eukaryotic expression systems. To improve recombinant AW-YGL production, six ER-associated factors, including HAC1, CPR5, PDI, ERO1, BIP, and AFT1, were individually co-expressed in the GS115/HSS-AW-YGL strain. PCR verification showed that the expression cassettes encoding the different chaperone-related factors were successfully integrated into the *P. pastoris* genome (Figure 6A).

The effects of different co-expressed factors on AW-YGL production were evaluated by measuring enzymatic activity, purified protein concentration, and specific activity. Among the tested factors, BIP co-expression showed the strongest enhancement effect. The GS115/HSS-AW-YGL-BIP strain exhibited an enzymatic activity of 6.1 × 10^5^ U/L, which was approximately 52.8% higher than that of the control strain (4.0 × 10^5^ U/L, **** *p* < 0.0001) (Figure 6B).

In comparison, the other co-expressed factors showed different effects on AW-YGL production but did not exhibit statistically significant improvement compared with the control strain. The enzymatic activities of ERO1-, CPR5-, PDI-, HAC1-, and AFT1-co-expressing strains were 5.0 × 10^5^, 4.6 × 10^5^, 3.7 × 10^5^, 4.2 × 10^5^, and 3.5 × 10^5^ U/L, respectively (Figure 6B).

Consistent with the activity results, BIP co-expression increased the purified recombinant protein concentration to 389.7 mg/L, compared with 330.7 mg/L in the control strain, representing an approximately 17.8% increase (**** *p* < 0.0001) (Figure 6C). The other chaperone co-expression strains showed protein concentrations ranging from 315.0 to 353.7 mg/L, without significant improvement compared with the control strain.

The specific activity of purified recombinant AW-YGL was further calculated based on enzymatic activity and protein concentration. The BIP co-expression strain exhibited a specific activity of 1560.3 U/mg, which was approximately 28.9% higher than that of the control strain (1264.2 U/mg, * *p* < 0.05) (Figure 6D). Other co-expression strains showed comparable or lower specific activities.

SDS-PAGE analysis revealed an apparent protein band around 15 kDa in all recombinant strains, with a stronger band intensity observed in the BIP co-expression strain (Figure 6E).

BIP was therefore identified as the key molecular chaperone for promoting high-level AW-YGL expression.

### 2.5. CRISPR/Cas9-Mediated Knockout of MNN1 and FKS1 Further Optimizes AW-YGL Expression

Cell wall-related engineering strategies were further investigated to improve recombinant AW-YGL production. Based on their reported roles in cell wall structure and glycosylation-related processes, MNN1 and FKS1 were selected as candidate genes for deletion in the GS115/HSS-AW-YGL-BIP strain.

**Figure 7:**
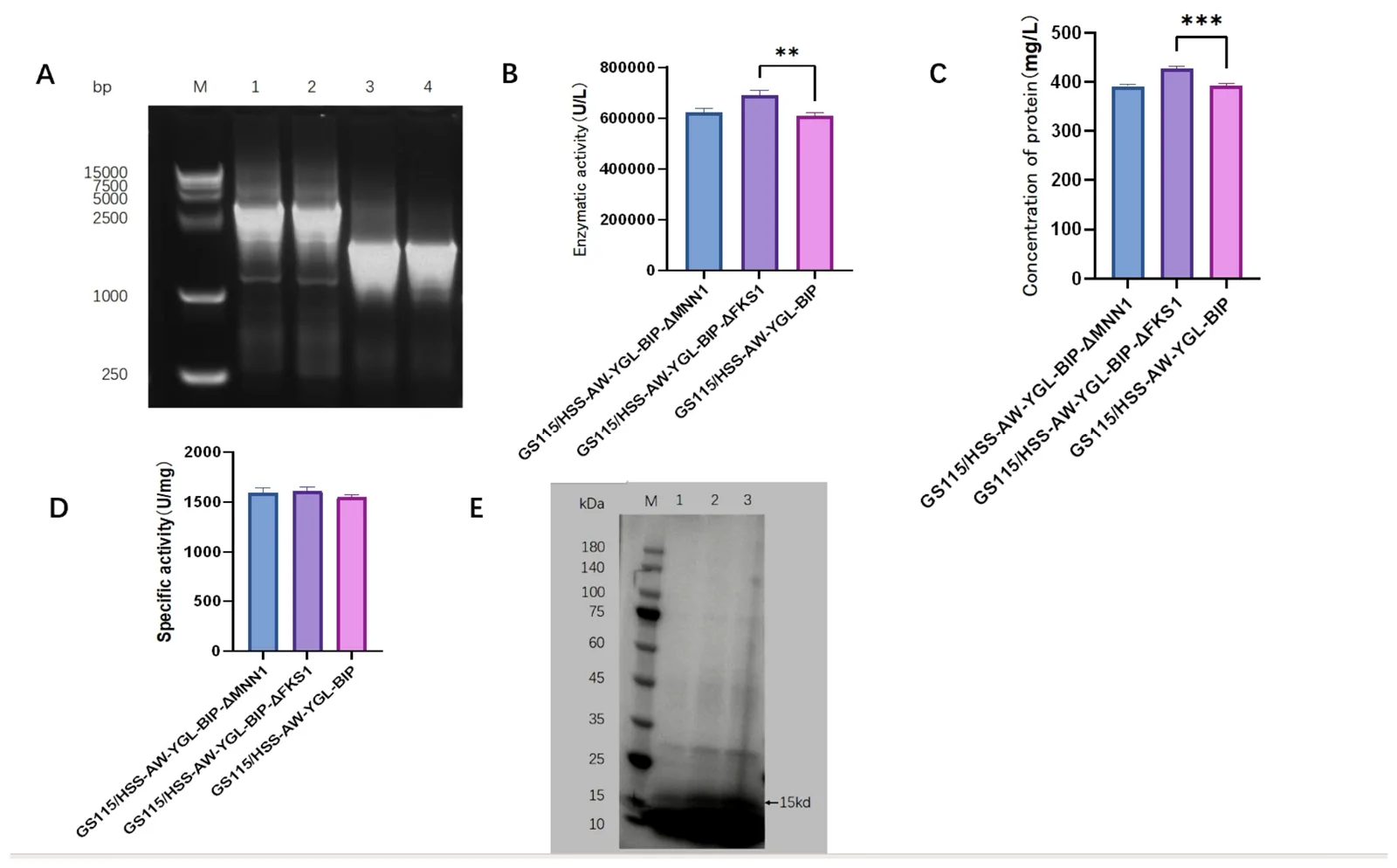
Effects of MNN1 and FKS1 knockout on AW-YGL secretory expression. (**A**) polymerase chain reaction (PCR) identification of MNN1 and FKS1 knockout strains. M, DNA molecular-weight marker; lane 1, before MNN1 knockout; lane 2, after MNN1 knockout; lane 3, before FKS1 knockout; lane 4, after FKS1 knockout. (**B**) Total AW-YGL enzyme activity in the fermentation supernatants of the different strains. (**C**) Total AW-YGL concentration after purification in the fermentation supernatants. (**D**) Specific activity of AW-YGL. (**E**) sodium dodecyl sulfate-polyacrylamide gel electrophoresis (SDS-PAGE) analysis of proteins in fermentation supernatants. M, protein molecular-weight marker; lane 1, GS115/HSS-AW-YGL-BIP; lane 2, GS115/HSS-AW-YGL-BIP-ΔMNN1; lane 3, GS115/HSS-AW-YGL-BIP-ΔFKS1. The arrow indicates the AW-YGL target protein at approximately 15 kDa. ** *p* < 0.01, *** *p* < 0.001.

PCR analysis was performed to evaluate the integration of the corresponding deletion constructs (Figure 7A). The effects of MNN1 and FKS1 deletion on AW-YGL production were subsequently assessed by enzymatic activity, purified protein concentration, and specific activity analysis.

Compared with the parental GS115/HSS-AW-YGL-BIP strain, deletion of MNN1 resulted in a slight increase in extracellular enzymatic activity, from 6.1 × 10^5^ U/L to 6.2 × 10^5^ U/L, whereas FKS1 deletion further increased the activity to 6.9 × 10^5^ U/L (Figure 7B). The FKS1 deletion strain exhibited an approximately 13.1% increase in total enzymatic activity compared with the parental strain.

Consistent with the activity results, purified recombinant protein concentration was slightly increased in the FKS1 deletion strain (427.3 mg/L) compared with the parental strain (392.0 mg/L, approximately 9.0% increase) (Figure 7C). In contrast, MNN1 deletion showed little effect on purified protein concentration (391.3 mg/L).

The specific activities of recombinant AW-YGL from the ΔMNN1 and ΔFKS1 strains were comparable to that of the parental strain. The specific activities were 1593.2 U/mg, 1612.6 U/mg, and 1638.3 U/mg for ΔMNN1, ΔFKS1, and parental strains, respectively (Figure 7D). These results suggest that FKS1 deletion mainly affected recombinant protein accumulation rather than the intrinsic catalytic activity of AW-YGL.

SDS-PAGE analysis showed apparent protein bands around 15 kDa in all recombinant strains (Figure 7E).

Thus, FKS1 knockout was associated with increased extracellular AW-YGL levels under the tested conditions, although the underlying mechanism requires further investigation.

### 2.6. Physicochemical Properties of Recombinant AW-YGL

To evaluate the application potential of the optimized AW-YGL protein, purified AW-YGL was analyzed for metal-ion stability, protease resistance, pH stability, and thermal stability.

#### 2.6.1. Thermal Stability

**Figure 8:**
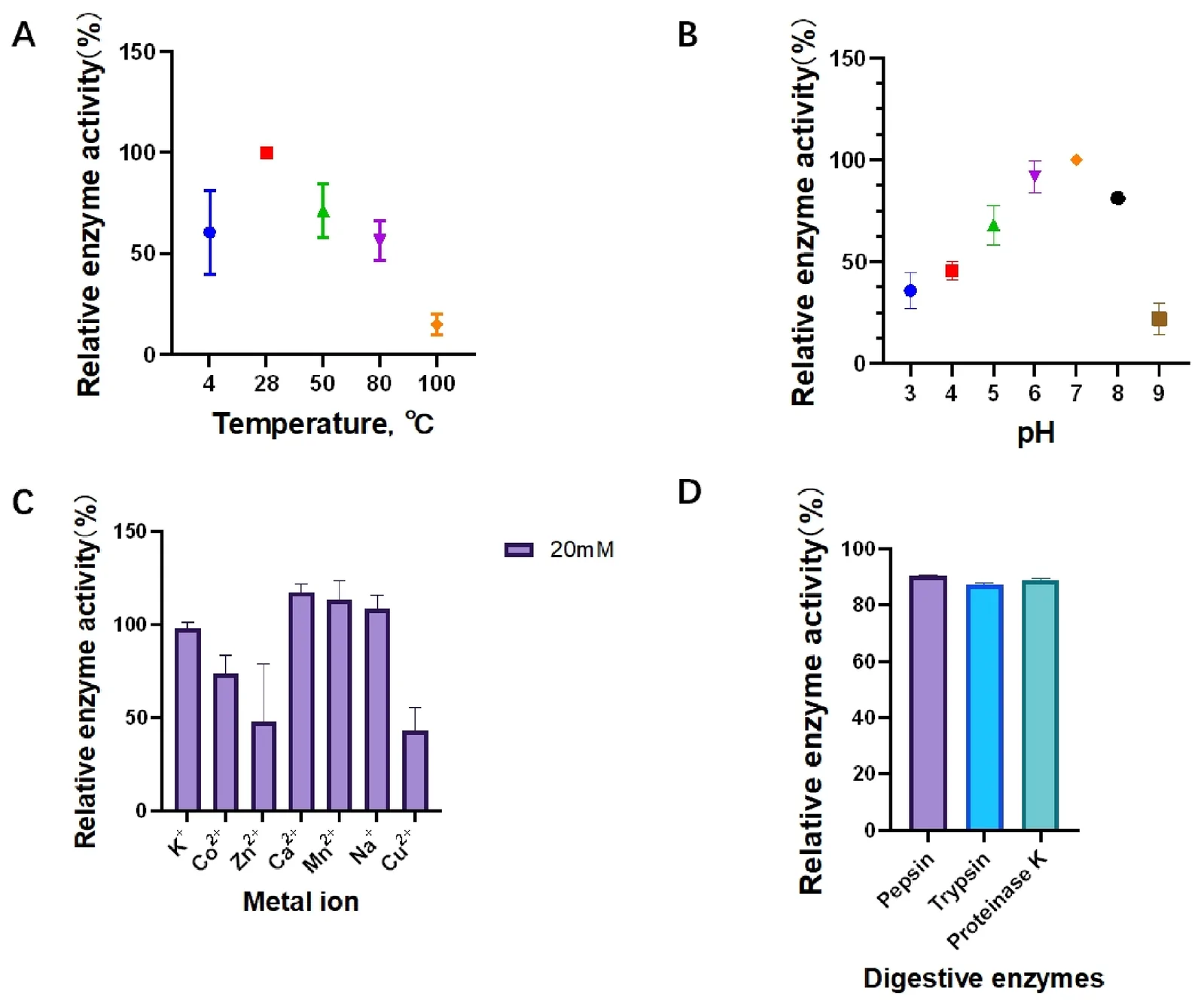
Effects of temperature and pH on the enzymatic stability of AW-YGL. (**A**) Effect of temperature on AW-YGL activity. Purified AW-YGL was incubated at 4, 28, 50, 80, and 100 °C for 30 min, followed by activity measurement under standard assay conditions. The residual activity of untreated AW-YGL was defined as 100%. AW-YGL retained measurable activity after temperature treatment, with relatively high residual activity observed at moderate temperatures. (**B**) Effect of pH on AW-YGL activity. Purified AW-YGL was incubated in Britton–Robinson buffers ranging from pH 3.0 to 9.0 for 30 min, and residual activity was determined under standard assay conditions. The residual activity of untreated AW-YGL was defined as 100%. AW-YGL maintained activity over a broad pH range, indicating tolerance to different pH conditions under the experimental conditions. (**C**) Effects of different metal ions on AW-YGL activity. Residual AW-YGL activity was measured in the presence of 20 mM K^+^, Co^2+^, Zn^2+^, Ca^2+^, Mn^2+^, Na^+^, or Cu^2+^, with the no-metal-ion group used as the control. Different metal ions affected AW-YGL activity to varying degrees. Ca^2+^, Mn^2+^, and Na^+^ modestly enhanced activity, whereas Cu^2+^ produced a pronounced inhibitory effect. (**D**) Stability of AW-YGL toward digestion-related proteases. Residual AW-YGL activity was measured after treatment with pepsin, trypsin, or proteinase K. AW-YGL retained relatively high activity after all protease treatments, indicating favorable protease resistance. Data are presented as mean ± SD (*n* = 3).

Temperature stability analysis showed that AW-YGL maintained high activity at moderate temperatures. The relative activity of untreated AW-YGL was defined as 100%. After incubation at 4, 50, 80, and 100 °C for 30 min, AW-YGL retained 60.6%, 71.2%, 56.5%, and 15.2% residual activity, respectively. Although enzyme activity decreased after exposure to elevated temperatures, AW-YGL maintained measurable activity even after treatment at 80 °C, whereas severe activity loss was observed at 100 °C. These results indicate that AW-YGL possesses moderate thermal stability under the tested conditions (Figure 8A).

#### 2.6.2. pH Stability

AW-YGL exhibited broad pH tolerance ranging from acidic to alkaline conditions. The activity of untreated AW-YGL was defined as 100%. After incubation under different pH conditions, AW-YGL retained 36.0%, 46.0%, 68.1%, 91.8%, 81.3%, and 22.0% residual activity at pH 3, 4, 5, 6, 8, and 9, respectively. The highest stability was observed near neutral conditions, with AW-YGL maintaining more than 80% activity between pH 6 and pH 8. These results suggest that AW-YGL exhibits favorable tolerance under near-neutral pH conditions (Figure 8B).

#### 2.6.3. Metal-Ion Stability

AW-YGL exhibited different responses to various metal ions. Compared with the untreated control (100%), Ca^2+^ and Mn^2+^ enhanced the relative enzymatic activity to 117.2% and 113.1%, respectively. In contrast, Cu^2+^ and Zn^2+^ decreased the activity to 42.9% and 48.2%, respectively. K^+^ and Na^+^ showed limited effects, maintaining relative activities of 98.1% and 108.7%, while Co^2+^ resulted in a moderate reduction of activity (73.9%). These results suggest that AW-YGL possesses differential metal ion tolerance under the tested conditions (Figure 8C).

#### 2.6.4. Protease Stability

Protease stability analysis showed that AW-YGL retained high residual enzymatic activity after treatment with different digestive enzymes. After incubation with pepsin, trypsin, and proteinase K, AW-YGL retained 90.5%, 87.2%, and 89.0% of its original activity, respectively. Among the tested proteases, pepsin treatment resulted in the highest residual activity, suggesting that AW-YGL maintained substantial stability under simulated gastric digestion conditions (Figure 8D).

## 3. Discussion

In this study, yak gastric lysozyme was first functionally engineered by fusing hydrophobic short peptides to its C-terminus. All four fusion proteins retained relatively high catalytic activity, but their antibacterial phenotypes did not fully parallel either enzyme activity or peptide hydrophobicity. AW-YGL showed the most pronounced activity against Gram-negative bacteria, with inhibition rates of approximately 65%, 68%, and 67% against *E. coli*, *P. aeruginosa*, and *S. enteritidis*, respectively. This result is consistent with previous approaches that broadened the antibacterial spectrum of lysozyme by increasing surface hydrophobicity or introducing functional peptide segments. For example, Ito et al. attached polyproline chains of different lengths to the C-terminus of human lysozyme and conferred antibacterial inhibition activity against Gram-negative bacteria on a protein that had primarily targeted Gram-positive organisms; this activity increased with increasing fusion-protein hydrophobicity [13]. Xu et al. fused a serine-rich heptapeptide to the C-terminus of chicken lysozyme. Although the secretion level of the fusion protein was approximately 60% of that of wild-type lysozyme, its antibacterial inhibition activity against E. coli was clearly enhanced [14]. In addition, Liu et al. increased the surface hydrophobicity of hen egg-white lysozyme by reductive modification and reported an approximately 1-log increase in antibacterial activity against both *E. coli* and *S. enteritidis* [26]. Because those studies used colony counting, antibacterial indices, or other activity readouts, whereas the present study calculated inhibition rates from changes in OD600, absolute values should not be directly compared across studies. Nevertheless, the collective evidence supports the general concept that altering the surface physicochemical properties of lysozyme can enhance its activity against Gram-negative bacteria.

Notably, AWVAWK was not the most hydrophobic of the four candidate peptides, yet it produced the strongest anti-Gram-negative phenotype, indicating that functional enhancement cannot be explained simply by a ‘higher hydrophobicity is better’ relationship. AWVAWK contains hydrophobic residues such as Ala and Val, two aromatic Trp residues, and positively charged Lys residues. The AWVAWK hexapeptide previously identified from porcine lysozyme itself exhibits activity against Gram-negative bacteria and has been proposed to act, at least in part, through membrane penetration [12]. Together with previous studies of cationic/hydrophobic antimicrobial peptides and hydrophobically modified lysozymes [9,10,11,13], our findings suggest that AWVAWK may enhance contact between AW-YGL and the Gram-negative outer membrane through electrostatic attraction and hydrophobic interactions, thereby increasing the opportunity for YGL to reach the peptidoglycan layer. However, outer-membrane permeability, membrane potential, and membrane integrity were not directly measured in the present study. Therefore, this mechanism remains hypothetical and should be evaluated using assays such as NPN uptake, propidium iodide staining, membrane-potential measurements, or microscopic analysis.

After AW-YGL was selected, its extracellular expression was optimized at the levels of host background and secretory entry. Replacing X-33 with GS115 increased total enzyme activity in the fermentation supernatant from 1.9 × 10^5^ U/L to 3.1 × 10^5^ U/L, an increase of approximately 63%, while specific activity increased from 821 U/mg to 983 U/mg, or approximately 20%. The higher expression level of AW-YGL in GS115 than in X-33 may be related to differences in their genetic backgrounds and protein-processing capacities. GS115 may provide a more favorable environment for recombinant protein folding and secretion, while reducing protein degradation, thereby promoting the accumulation of functional AW-YGL.Further replacement of the secretion signal with HSS in the GS115 background increased total enzyme activity to 4.3 × 10^5^ U/L, approximately 39% above that of the original GS115/AW-YGL strain, and increased specific activity from 983 U/mg to 1264 U/mg, or approximately 29%. These results indicate that, for the same target protein, both host genetic background and compatibility between the signal peptide and the cargo protein can substantially affect the extracellular accumulation of functional product. The increased specific activity observed after signal peptide optimization may not simply result from enhanced protein secretion, but may also reflect improved processing and maturation of recombinant AW-YGL. Signal peptides can influence the efficiency of translocation into the endoplasmic reticulum (ER), N-terminal processing, and subsequent folding of secreted proteins in *P. pastoris*. Different signal peptides may affect the proportion of correctly folded and biologically active proteins in the extracellular environment. Therefore, although the total protein yield may not always increase substantially, optimization of the secretion pathway may improve the quality and functional proportion of the secreted recombinant protein, resulting in increased specific activity. These findings further support empirical screening of signal peptides for individual target proteins rather than reliance on a single universally optimal secretion signal.

This signal-peptide dependence agrees with previous findings in *P. pastoris*. Duan et al. screened endogenous signal peptides and found that the Msb2 signal peptide increased the extracellular specific yields of three reporter proteins by 1.5- to 8.0-fold, with markedly different magnitudes among target proteins [22]. Other studies have likewise demonstrated strong cargo-specificity in signal-peptide performance [27,28]. In the present study, HSS increased AW-YGL total enzyme activity by approximately 1.39-fold. Although this increase was smaller than that observed for some highly responsive reporter proteins, it was accompanied by increased specific activity, indicating that HSS optimization enhanced extracellular accumulation of functional protein without an obvious loss of activity.

On the basis of HSS optimization, co-expression of ER-associated factors further improved AW-YGL production, with BIP producing the most pronounced effect. BIP co-expression increased total enzyme activity from 4.0 × 10^5^ U/L to approximately 6.1 × 10^5^ U/L, an increase of about 40%, and increased specific activity from 1264 U/mg to 1560 U/mg, or approximately 23%. This magnitude of improvement is comparable to that reported for folding/UPR engineering in other systems. Liu et al. co-expressed Hac1p in a four-copy human lysozyme strain and increased extracellular AW-YGL concentration after purification from 436.99 μg/mL to 517.82 μg/mL and enzyme activity from 61,900 U/mL to 78,600 U/mL, corresponding to increases of approximately 19% and 27%, respectively [23]. However, the effects of molecular chaperones are not universal. He et al. reported that co-expression of Pdi1 or Ero1 enhanced secretion in a human lysozyme system, whereas BIP co-expression did not increase secretion and reduced enzyme activity from 14,680 U/mL to 10,200 U/mL [29]. In contrast, BIP exerted a clear positive effect on AW-YGL in the present study, highlighting the cargo dependence of folding bottlenecks and suggesting that AWVAWK fusion may alter the folding or ER quality-control requirements of YGL compared with unmodified human lysozyme.

CRISPR/Cas9 was subsequently used to engineer the host by deleting MNN1 and FKS1. For MNN1, the most appropriate interpretation is limited to the possibility that the mannosylation pathway affects secreted-protein processing. In this study, FKS1 deletion increased extracellular AW-YGL production while maintaining a comparable specific activity to the parental strain, suggesting that the beneficial effect of FKS1 deletion was mainly associated with increased recombinant protein accumulation rather than changes in the intrinsic catalytic properties of AW-YGL.Previous studies have shown that yeast N-glycosylation and mannose-chain extension pathways can be remodeled through host engineering [30,31,32,33,34]. However, the present study did not directly analyze AW-YGL glycan composition, glycosylation-site occupancy, or molecular-weight changes. Therefore, the ΔMNN1 expression phenotype alone cannot support the conclusion that MNN1 deletion reduces abnormal glycosylation and improves protein quality. Future mechanistic studies should incorporate glycoprotein staining, PNGase F treatment, or mass-spectrometry-based glycomics.

In contrast, the relationship between FKS1 and cell-wall architecture is more direct. FKS1 encodes a key catalytic component of β-1,3-glucan synthase and is a central determinant of yeast cell-wall β-glucan formation [35,36]. In the GS115/HSS-AW-YGL-BIP background, FKS1 knockout increased total enzyme activity from approximately 6.1 × 10^5^ U/L to 6.9 × 10^5^ U/L, or about 13.1%, while specific activity increased from 1593.2 U/mg to 1638.3 U/mg. These results suggest that the major contribution of this step may be improved extracellular protein accumulation rather than a substantial alteration of AW-YGL catalytic efficiency. Indirect evidence supports the feasibility of cell-wall engineering as a strategy to improve secretion. Offei et al. reported that inactivation of the mannosyltransferase HOC1 in Komagataella phaffii weakened the cell wall and increased β-glucosidase secretion by approximately twofold [37], while studies involving GAS1 and cell-wall stress responses have also shown that cell-wall remodeling can alter secretion phenotypes [38,39].

Considering the entire optimization process, total AW-YGL activity in the fermentation supernatant increased from 1.9 × 10^5^ U/L in the initial X-33 system to 6.9 × 10^5^ U/L in the final ΔFKS1 strain, representing an overall increase of approximately 3.6-fold. Specific activity increased from 821 U/mg to 1638.3 U/mg, or approximately 2.1-fold. FKS1 deletion may enhance recombinant AW-YGL production by modifying the cellular environment associated with cell wall formation and protein secretion. Fks1 is involved in β-glucan synthesis and cell wall organization, and alterations in cell wall architecture may influence secretion efficiency or extracellular release of recombinant proteins. However, these studies do not directly establish that FKS1 deficiency necessarily enhances recombinant-protein secretion. The present findings are therefore more appropriately viewed as experimental evidence identifying FKS1 as a potential host-engineering target. The underlying mechanism should be examined by measuring cell-wall β-glucan content, wall thickness, permeability, and growth characteristics. This cumulative improvement is consistent with the general principle that multilevel engineering can outperform a single intervention in *P. pastoris*. For example, Wang et al. combined high-expression clone screening, signal-sequence optimization, VPS10 disruption, and molecular-chaperone/transcription-factor co-expression to increase human lysozyme activity in shake-flask culture to approximately fivefold that of the original strain [21]. Jia et al. also achieved an approximately twofold increase in human lysozyme activity through fermentation-process control [20]. It should be emphasized that lysozyme source, culture mode, substrate, and the definition of enzyme-activity units differ among studies; therefore, absolute activity values should not be compared directly, and relative fold improvements provide a more appropriate basis for evaluating engineering effectiveness.

Stability analysis showed that the optimized AW-YGL retained measurable residual activity across defined pH and temperature ranges and showed tolerance to several metal ions and digestion-related proteases. This behavior is consistent with the biological background of gastric lysozymes, which have evolved under acidic and protease-rich conditions [4,5,40]. Nevertheless, the present stability assays primarily reflect residual enzyme activity after short-term treatment and should not be interpreted directly as storage stability, processing stability, or in vivo efficacy. Accordingly, the principal contribution of this study is the establishment of a sequential optimization route integrating functional peptide engineering, host/signal-peptide screening, ER folding assistance, and cell-wall engineering. This strategy enhanced both the inhibitory activity of YGL against Gram-negative bacteria and its secretory expression in *P. pastoris*. Future work should further investigate the membrane-interaction mechanism, changes in cell-wall structure, scale-up fermentation performance, long-term stability, and safety to determine the translational potential of AW-YGL in food, feed, or other antimicrobial applications.

## 4. Materials and Methods

### 4.1. Strains, Plasmids, Genes, and Reagents

The *P. pastoris* strains X-33 and GS115 used in this study were provided by the research group of Yunhe Cao at the Feed Engineering Center, China Agricultural University. The expression vector pGAPZαA and the CRISPR/Cas9 gene-editing vector pPpT4-pHTX1-hsCas9 were purchased from Beijing Liuhe BGI Technology Co., Ltd. (Beijing, China) *Escherichia coli* (ATCC 25922), *Staphylococcus aureus* (ATCC 6538), *Salmonella enteritidis* (ATCC 10428), and *Pseudomonas aeruginosa* (ATCC 9027) were purchased from Beijing Bio-Collection Biotechnology Co., Ltd. (Beijing, China) Chemically competent E. coli TOP10 cells were purchased from Tsingke Biotechnology Co., Ltd. (Beijing, China).

The yak gastric lysozyme gene (AMR70500.1) and the *P. pastoris* functional genes HAC1 (PAES_05430), CPR5 (PAS_chr4_0138), PDI (PAS_chr4_0217), ERO1 (PAS_chr1_0464), BIP (PAS_chr2_0701), and AFT1 (PAS_chr3_0981) were synthesized by Beijing Liuhe BGI Technology Co., Ltd. (Beijing, China) All experimental primers were synthesized by Vazyme Biotech Co., Ltd. (Nanjing, China).

The antibiotics used in this study, including Zeocin, kanamycin, G418, ampicillin, and nourseothricin, were purchased from Beijing Solarbio Science & Technology Co., Ltd. (Beijing, China) Yeast extract, tryptone, glucose, and agar used for culture media were purchased from Oxoid (Basingstoke, UK). The homologous recombination kit was purchased from Sino-United Biotechnology Co., Ltd. (Beijing, China), and the restriction endonuclease DraI was purchased from Beijing Baiwang Tongda Technology Co., Ltd. (Beijing, China) Agarose-gel DNA recovery, PCR-product purification, and plasmid extraction kits were purchased from Sigma-Aldrich (Shanghai) Trading Co., Ltd. (Shanghai, China) The SDS-PAGE kit was obtained from Changzhou Boyi Biotechnology Co., Ltd. (Changzhou, China), the BCA protein assay kit from Beyotime Biotechnology (Shanghai, China), and the lysozyme activity assay kit from Suzhou Grace Biotechnology Co., Ltd. (Suzhou, China).

### 4.2. Synthesis of Modified Yak Gastric Lysozyme Genes

Four hydrophobic peptides (AWVAWK, FFVAP, GVSLANWVCLAK, and VVYP) were selected based on previously reported functional peptide sequences. AWVAWK and FFVAP were derived from Zhang Lizhi’s study on the heterologous expression and antibacterial activity of human lysozyme, GVSLANWVCLAK was obtained from Zhu Dewei’s study on recombinant porcine lysozyme enhancement, and VVYP was selected from Zhao Mouming’s review on food-derived bioactive peptides. These peptides were selected due to their different hydrophobic characteristics and potential functional effects. The designed coding sequences were as follows: FF (TTTTTCGTTGCTCCA), GV (GGTGTTTCTTTGGCTAACTGGGTTTGTTTGGCTAAG), VV (GTTGTTTACCCA), and AW (GCTTGGGTTGCTTGGAAG). The sequences and physicochemical properties of the four hydrophobic peptides are shown in Table 1.

Hydrophobicity values were calculated according to the Kyte–Doolittle scale, which assigns a specific hydropathy index to each amino acid residue based on its relative hydrophobicity. The average hydrophobicity value of each peptide was calculated as the mean hydropathy index of all amino acid residues within the peptide sequence.

The yak gastric lysozyme (YGL) gene sequence was retrieved from the National Center for Biotechnology Information (NCBI) database (Bethesda, MD, USA; https://www.ncbi.nlm.nih.gov/; accessed on 20 October 2026). Based on the codon preference of the *P. pastoris* expression system, the target gene was codon-optimized using SnapGene (version 6.0.2; GSL Biotech LLC, Boston, MA, USA). The optimized YGL gene was synthesized by Beijing Liuhe BGI Biotechnology Co., Ltd. (Beijing, China).

To construct hydrophobic-peptide-fused yak gastric lysozyme genes, specific primers were designed to amplify fusion fragments containing the coding sequence of each target peptide. The four hydrophobic peptide coding sequences were individually fused to the carboxyl terminus (C-terminus) of the YGL gene to generate modified yak gastric lysozyme genes (mYGL). The recombinant plasmid contained the α-factor secretion signal sequence, the peptide-YGL coding region, and the C-terminal His tag for subsequent protein purification The resulting gene fragments were sequence-verified and used for subsequent expression-vector construction.

### 4.3. Construction and Transformation of Recombinant Expression Vectors

Recombinant expression vectors were constructed by homologous recombination. According to the experimental design, the purified lysozyme gene fragment was mixed with the linearized expression-vector fragment at the appropriate ratio and assembled using a homologous recombination enzyme mix (Sino-United Biotechnology Co., Ltd., Beijing, China). The resulting recombinant plasmids were transformed into chemically competent Escherichia coli TOP10 cells. Briefly, 10 μL of recombinant plasmid was mixed with 50 μL of TOP10 competent cells, incubated on ice, heat-shocked at 42 °C for 60 s, and immediately cooled on ice for 2 min. Subsequently, 700 μL of SOC medium was added, and the cells were recovered at 37 °C and 200 rpm for 2 h. The recovered culture was concentrated by centrifugation at 3000 rpm for 3 min. After removal of part of the supernatant, the cells were resuspended and spread evenly on LB agar plates containing Zeocin (100 μg/mL), followed by incubation at 37 °C to select positive transformants. Single colonies were further verified by colony PCR and Sanger sequencing to confirm correct recombinant-vector construction.

### 4.4. Heterologous Expression of Recombinant Vectors in Pichia pastoris

Recombinant expression plasmids were linearized with the restriction endonuclease DraI. After incubation at 37 °C for 30 min, the linearized DNA was purified using a PCR-product purification kit. Ten microliters of the purified linearized recombinant plasmid was then added to competent *P. pastoris* cells, gently mixed on ice, and allowed to stand for 10 min. The recombinant plasmid was introduced into *P. pastoris* by electroporation at 1.5 kV with a pulse time of 5–7 ms. Immediately after electroporation, 1 mL of pre-chilled sorbitol solution was added. The cells were gently mixed, transferred to a sterile centrifuge tube, and recovered at 30 °C and 200 rpm for 4 h.

The recovered transformation mixture was concentrated by centrifugation at 3000 rpm for 3 min. After removal of part of the supernatant, the cells were resuspended and spread evenly on YPD agar plates containing Zeocin (100 μg/mL), followed by inverted incubation at 30 °C until single colonies formed. Colonies were randomly selected for colony-PCR identification, and positive transformants carrying the recombinant expression vector were obtained.

Positive recombinant strains were inoculated into 200 mL of YPD liquid medium and cultured at 30 °C and 200 rpm for 120 h to produce recombinant AW-YGL for subsequent protein detection and purification.

### 4.5. Expression, Purification, and Identification of mYGL

#### 4.5.1. Expression and Enzymatic Activity Analysis of Recombinant mYGL

After recombinant strains were cultured in YPD liquid medium for 120 h, the fermentation broth was collected and centrifuged at 12,000 rpm for 10 min at 4 °C to remove the cells. The culture supernatant containing the secreted recombinant protein was collected. Because the pGAPZαA expression vector carries an α-factor secretion signal, recombinant AW-YGL could be secreted into the culture supernatant.

Lysozyme activity assay: Lysozyme activity was determined using the commercial lysozyme activity assay kit (G1207W, Solarbio Life Sciences, Beijing, China) according to the manufacturer’s instructions. Briefly, culture supernatant or purified recombinant mYGL samples were mixed with the corresponding reaction reagents, incubated under the recommended reaction conditions, and the absorbance was measured at 530 nm. A reagent blank and standard controls were included for calibration. Lysozyme activity was calculated from the change in absorbance (ΔA) according to the standard curve generated by the kit and expressed as enzyme activity units per milliliter (U/mL) or specific activity after normalization to protein concentration.

An appropriate volume of culture supernatant was analyzed by SDS-PAGE to detect recombinant mYGL expression. Lysozyme activity in the culture supernatant was measured using a lysozyme activity assay kit, and enzyme activity was calculated according to the manufacturer’s standard protocol.

#### 4.5.2. Purification of mYGL

His-tagged recombinant mYGL was purified by Ni^2+^-NTA affinity chromatography. The culture supernatant was first loaded onto a pre-equilibrated Ni^2+^-NTA affinity column to allow binding of the His-tagged recombinant protein to nickel ions. Nonspecifically bound proteins were then sequentially removed using elution buffers containing 20, 50, 100, and 200 mM imidazole. Finally, the target recombinant mYGL was eluted with buffer containing 500 mM imidazole. Fractions containing the target protein were collected and analyzed by SDS-PAGE to assess purity. Purified protein samples were subsequently dialyzed or desalted to remove imidazole.

#### 4.5.3. Protein Concentration Determination

The concentration of purified mYGL was determined using the bicinchoninic acid (BCA) assay. Bovine serum albumin (BSA) was used as the standard protein to prepare a series of standard solutions. Absorbance at 562 nm (A562) was measured to generate a standard curve. The concentration of purified mYGL was calculated from the standard curve and adjusted uniformly to 1 mg/mL for antibacterial inhibition assays.

### 4.6. Determination of the Antibacterial Inhibition Rate of mYGL

The antibacterial activity of recombinant AW-YGL against different Gram-positive and Gram-negative bacterial strains was evaluated using a 96-well microplate-based assay. Briefly, bacterial strains were cultured to the logarithmic growth phase, and the cell suspensions were adjusted to an initial optical density at 600 nm (OD600) value of 0.05 using phosphate-buffered saline (PBS).

Purified recombinant AW-YGL was dissolved in protein-storage buffer at a concentration of 1 mg/mL. For antibacterial analysis, 10 μL of bacterial suspension was mixed with 190 μL of AW-YGL solution in each well of a 96-well microplate, resulting in a final reaction volume of 200 μL and a final AW-YGL concentration of 0.95 mg/mL. For the negative control group, an equal volume of protein-storage buffer was added instead of recombinant AW-YGL.

The reaction mixtures were incubated at 37 °C for 12 h, and OD600 values were measured at 0 h and 12 h. The antibacterial activity was evaluated based on the reduction in OD600 compared with the negative control group. All experiments were performed using three independent biological replicates.

The antibacterial inhibition rate of mYGL against each strain was calculated from the change in bacterial turbidity during culture using the following equation:

Inhibition rate (%) = [1 − (ODtreatment,12 h − ODtreatment,0 h)/(ODcontrol,12 h − ODcontrol,0 h)] × 100%.

### 4.7. Effects of Different Pichia pastoris Hosts on AW-YGL Expression

The recombinant plasmid pGAPZα-AW-YGL was transformed separately into *P. pastoris* GS115 and X-33. Positive recombinant strains were identified by PCR and designated GS115/AW-YGL and X-33/AW-YGL, respectively. Recombinant protein expression was performed under identical culture conditions, and fermentation supernatants were collected at the end of cultivation. Lysozyme activity in the supernatant was determined using a lysozyme activity assay kit according to the manufacturer’s standard protocol. His-tagged recombinant AW-YGL was purified by Ni2+-NTA affinity chromatography, and total AW-YGL concentration after purification was measured by the BCA method. Specific activity was calculated from enzyme activity and AW-YGL concentration after purification. AW-YGL expression was also analyzed by SDS-PAGE.

### 4.8. Signal-Peptide Optimization of Recombinant Plasmids

To improve the secretory expression of recombinant AW-YGL, the expression vector was optimized by replacing the secretion signal with different signal-peptide sequences. Specific primers containing vector-homology arms were used to amplify AW-YGL fragments fused to the HSS, ISS, YS, Exg1, or Msb2 signal peptide. The signal peptide was directly fused to the N-terminus of YGL without an additional linker sequence. Thus, no exogenous linker residues were introduced between the signal peptide and YGL.The corresponding linearized expression-vector fragments were amplified, and each signal-peptide-fused AW-YGL fragment was assembled with the linearized vector by homologous recombination to generate signal-peptide-optimized recombinant expression plasmids. All recombinant plasmids were verified by PCR and sequencing before subsequent *P. pastoris* transformation and expression analysis.

### 4.9. Co-Expression of Molecular Chaperones

Gene sequences encoding ER-associated factors in *P. pastoris*, including CPR5, BIP, PDI, ERO1, AFT1, and HAC1, were obtained from the NCBI database. The target sequences were optimized according to *P. pastoris* codon preference, and the optimized genes, together with a G418 selection marker, were synthesized by Beijing Liuhe BGI Technology Co., Ltd. (Beijing, China).

Each optimized gene, together with a G418 selection marker, was cloned into the pGAPZα expression vector under the control of the constitutive GAP promoter to generate the corresponding molecular chaperone expression plasmids. The recombinant plasmids were verified by PCR and sequencing, linearized with DraI, and introduced into competent *P. pastoris* cells by electroporation. Transformants were selected on YPD agar plates containing G418 (200 μg/mL), and positive colonies were identified by yeast colony PCR for subsequent molecular chaperone co-expression analysis.

### 4.10. Construction of CRISPR/Cas9 Vectors for MNN1 and FKS1 Knockout

Specific single-guide RNA (sgRNA) targets were designed for MNN1 (XP_002492593) and FKS1 (8196641) based on the *P. pastoris* genome sequence. Using the CRISPR/Cas9 editing vector pPpT4-pHTX1-hsCas9 as the template, primer pairs MNN1-1-F/MNN1-1-R, MNN1-2-F/MNN1-2-R, FKS1-1-F/FKS1-1-R, and FKS1-2-F/FKS1-2-R were used to amplify DNA fragments containing the corresponding sgRNA expression elements (MNN1-1, MNN1-2, FKS1-1, and FKS1-2).

The sgRNA expression fragments were inserted individually into pPpT4-pHTX1-hsCas9 by homologous recombination to construct CRISPR/Cas9 knockout plasmids targeting MNN1 and FKS1. Recombinant plasmids were transformed into competent E. coli TOP10 cells and spread on LB agar plates containing nourseothricin (NTC, 100 μg/mL) to select positive clones. Plasmids were extracted from positive clones for subsequent *P. pastoris* genome-editing experiments.

#### 4.10.1. Construction of Homologous Repair Templates (Donor DNA)

Genomic DNA from *P. pastoris* GS115 was extracted as the template. Based on the MNN1 and FKS1 sequences in the NCBI database, 1000-bp upstream and downstream homology arms were obtained for each target gene. Primer pairs MNN1-Donor1-F/MNN1-Donor1-R and MNN1-Donor2-F/MNN1-Donor2-R, as well as FKS1-Donor1-F/FKS1-Donor1-R and FKS1-Donor2-F/FKS1-Donor2-R, were used to amplify the corresponding upstream and downstream homology arms. The fragments were assembled by overlap-extension PCR to generate approximately 2000-bp homologous repair templates for MNN1 and FKS1 (Donor-MNN1 and Donor-FKS1).

#### 4.10.2. CRISPR/Cas9-Mediated Gene Knockout and Screening of Positive *Pichia pastoris* Strains

One hundred nanograms of circular pPpT4-pHTX1-hsCas9-MNN1 knockout plasmid and 200 ng of Donor-MNN1 repair template were co-electroporated into competent *P. pastoris* cells. In parallel, 100 ng of circular pPpT4-pHTX1-hsCas9-FKS1 knockout plasmid and 200 ng of Donor-FKS1 repair template were co-transformed into *P. pastoris* cells. Transformants were spread on YPD agar plates containing nourseothricin (NTC, 100 μg/mL) and incubated at 30 °C to obtain resistant colonies.

Resistant colonies were randomly selected for genotyping to identify MNN1- and FKS1-knockout strains. To remove the CRISPR/Cas9 editing plasmid, positive knockout strains were serially passaged on NTC-containing plates and subsequently transferred to nonselective YPD plates for continuous cultivation. Loss of the editing plasmid was confirmed by the inability of the strains to regrow on NTC-containing plates. Primers MNN1-1-F/MNN1-2-R and FKS1-1-F/FKS1-2-R, designed approximately 1000 bp upstream and downstream of the target knockout loci, were used for PCR verification. Positive clones were used for subsequent expression analysis.

### 4.11. Physicochemical Characterization of Recombinant AW-YGL

Purified recombinant AW-YGL was diluted with the appropriate buffer to a final concentration of 1 mg/mL for subsequent stability analyses.

#### 4.11.1. Metal-Ion Stability Analysis

Solutions of 40 mM KCl, NaCl, CaCl_2_, MnCl_2_, CoCl_2_, ZnCl_2_, and CuCl_2_ were prepared in 50 mM Tris-HCl buffer (pH 7.0). Each metal-ion solution was mixed with purified AW-YGL (1 mg/mL) at a 1:1 (*v*/*v*) ratio, giving a final metal-ion concentration of 20 mM. The mixtures were incubated at room temperature for 30 min, after which residual lysozyme activity was measured. An equal volume of 50 mM Tris-HCl buffer (pH 7.0) was used instead of the metal-ion solution for the control, and its relative activity was defined as 100%.

#### 4.11.2. Protease Stability Analysis

Pepsin, trypsin, and proteinase K solutions were prepared using different buffer systems. Pepsin was prepared in 50 mM glycine-HCl buffer (pH 2.0), trypsin in 50 mM Tris-HCl buffer (pH 8.0), and proteinase K in 50 mM Tris-HCl buffer (pH 7.5).

Purified AW-YGL (1 mg/mL) was mixed separately with each protease solution at an enzyme-to-substrate mass ratio of 1:50 and incubated at 37 °C for 30 min. After incubation, reactions containing pepsin were terminated by adjusting the pH to neutrality with 1 mol/L Tris-HCl buffer (pH 8.0), whereas trypsin- and proteinase K-treated samples were terminated by adding phenylmethylsulfonyl fluoride (PMSF) to a final concentration of 1 mM. All samples were then adjusted to the standard conditions required for lysozyme activity measurement, and residual enzyme activity was determined. Controls were treated identically using the corresponding buffer in place of protease, and their relative activities were defined as 100%.

#### 4.11.3. pH Stability Analysis

Britton–Robinson buffers at pH 3.0, 4.0, 5.0, 6.0, 7.0, 8.0, and 9.0 were prepared. Purified AW-YGL at a final concentration of 1 mg/mL was incubated in the corresponding buffers at room temperature for 30 min. After treatment, the samples were adjusted to the standard reaction conditions required for lysozyme activity measurement, and residual activity was determined. Untreated AW-YGL was used as the control and its relative activity was defined as 100% to evaluate the effect of different pH conditions on AW-YGL stability.

#### 4.11.4. Temperature Stability Analysis

Purified AW-YGL (1 mg/mL) was incubated at 4, 28, 50, 80, or 100 °C for 30 min, immediately cooled in an ice bath, and then returned to the standard conditions for lysozyme activity measurement. Residual activity was measured, and untreated AW-YGL was used as the control with its relative activity defined as 100% to assess thermal stability.

### 4.12. Statistical Analysis

All experiments included at least three biological replicates, and data are presented as the mean ± standard deviation (mean ± SD). Statistical analyses and graph preparation were performed using GraphPad Prism 8.0 (GraphPad Software, Boston, MA, USA). Differences among experimental groups were analyzed by one-way or two-way analysis of variance (ANOVA), as appropriate, followed by Tukey’s multiple-comparisons test. A *p* value < 0.05 was considered statistically significant. Significance levels were defined as follows: *p* < 0.05 (*), *p* < 0.01 (**), *p* < 0.001 (***), and *p* < 0.0001 (****).

## 5. Conclusions

In this study, a *P. pastoris* secretory-expression system for the engineered yak gastric lysozyme AW-YGL was established and systematically optimized. Fusion of different hydrophobic short peptides to the YGL C-terminus demonstrated that hydrophobic/aromatic-residue composition can markedly influence inhibitory activity against Gram-negative bacteria. AWVAWK produced the strongest enhancement and improved the inhibitory effects of YGL against *E. coli*, *P. aeruginosa*, and *Salmonella* while preserving lysozyme catalytic activity. Subsequent host and secretion-pathway engineering showed that the GS115 host, HSS signal peptide, and BIP co-expression were associated with improved extracellular levels and functional activity of AW-YGL under the tested conditions. On this optimized background, FKS1 knockout increased total enzyme activity in the fermentation supernatant to 6.9 × 10^5^ U/L and specific activity to 1638.3 U/mg, suggesting that cell-wall-related host engineering may contribute to the improvement of AW-YGL production, although further investigation is required to clarify the underlying mechanism. The presence of additional bands in some SDS-PAGE profiles may indicate possible host-derived proteins or protein heterogeneity; however, further characterization by Western blotting or mass spectrometry is required to determine their identity. Physicochemical characterization showed that optimized AW-YGL retained tolerance to defined pH and temperature conditions, metal ions, and proteases. These results indicate that engineered AW-YGL exhibits enhanced antibacterial activity and improved enzymatic properties. However, further studies are required to evaluate its safety, long-term stability, potential resistance development, and fermentation scalability before practical applications in feed additives or industrial production.

## Figures and Tables

**Table 1:** Sequence information and physicochemical properties of hydrophobic peptides used in this study.

Name	Sequence	Length (AA)	Net Charge	Hydrophobicity
FF	FFVAP	5	0	2.00
GV	GVSLANWVCLAK	12	1	1.05
VV	VVYP	4	0	1.38
AW	AWVAWK	6	1	0.35

## Data Availability

The datasets generated and analyzed during the current study are included in this article and its Supplementary Materials. The original uncropped gel images generated during the experiments are no longer available. All figures and supporting data presented in the revised manuscript have been prepared from the available experimental records, and this limitation has been transparently disclosed.

## Supplementary Materials

The following supporting information can be downloaded at: https://www.mdpi.com/article/10.3390/ijms27219403/s1.
